# Recent Advances in the Synthesis and Biomedical Applications of Nanocomposite Hydrogels

**DOI:** 10.3390/pharmaceutics7040413

**Published:** 2015-10-13

**Authors:** Umile Gianfranco Spizzirri, Manuela Curcio, Giuseppe Cirillo, Tania Spataro, Orazio Vittorio, Nevio Picci, Silke Hampel, Francesca Iemma, Fiore Pasquale Nicoletta

**Affiliations:** 1 Department of Pharmacy, Health and Nutritional Sciences, University of Calabria, I-87036 Rende, Italy; E-Mails: g.spizzirri@unical.it (U.G.S.); manuela.curcio@unical.it (M.C.); tania_spataro@libero.it (T.S.); nevio.picci@unical.it (N.P.); francesca.iemma@unical.it (F.I.); fiore.nicoletta@unical.it (F.P.N.).; 2 Children’s Cancer Institute Australia, Lowy Cancer Research Centre, University of New South Wales, Sydney, 2052, Australia; E-Mail: OVittorio@ccia.org.au; 3 Australian Centre for Nanomedicine, University of New South Wales, Sydney, 2052, Australia; 4 Leibniz Institute for Solid State and Materials Research, PF 270116, D-01171 Dresden, Germany; E-Mail: s.hampel@ifw-dresden.de

**Keywords:** carbon nanotubes, graphene, composite hydrogels, electro-responsive

## Abstract

Hydrogels sensitive to electric current are usually made of polyelectrolytes and undergo erosion, swelling, de-swelling or bending in the presence of an applied electric field. The electrical conductivity of many polymeric materials used for the fabrication of biomedical devices is not high enough to achieve an effective modulation of the functional properties, and thus, the incorporation of conducting materials (e.g., carbon nanotubes and nanographene oxide) was proposed as a valuable approach to overcome this limitation. By coupling the biological and chemical features of both natural and synthetic polymers with the favourable properties of carbon nanostructures (e.g., cellular uptake, electromagnetic and magnetic behaviour), it is possible to produce highly versatile and effective nanocomposite materials. In the present review, the recent advances in the synthesis and biomedical applications of electro-responsive nanocomposite hydrogels are discussed.

## 1. Electro-Responsive Hybrid Hydrogels in Drug Delivery

Smart hydrogels are functional materials able to modulate equilibrium swelling as a function of a change in the external environment, such as pH, ionic strength, temperature, pressure, light, electro-magnetic and sound fields [1,2,3,4,5,6]. Over the last few decades, they have emerged as valuable tools for applications in biomedicine, mainly as drug delivery matrices and tissue engineering scaffolding materials [7,8].

An external electric field is a useful approach to activate/modulate the release of therapeutics, since the availability of equipment for controlling the magnitude of current, the duration of electric pulses, the intervals between pulses, *etc.*, allows the application of such a stimulus to be precisely handled [9,10].

The response of electro-responsive hydrogels to an external electric field can be erosion, swelling, de-swelling or actuation of the structure according to different mechanisms (Coulombic, electro-osmotic, electro-chemical and dynamic enrichment/depletion phenomena) [11] and to several parameters, including the experimental setup, the gel composition (charge density, swelling degree, nature of monomers, cross-linkers and pendant chains), pH, current magnitude, ionic strength, presence of drugs, *etc.* [12]. In particular, a low polyion concentration results in hydrogel de-swelling, while swelling is recorded at higher concentrations [13].

A key limitation for the preparation of effective electro-sensitive releasing devices is related to the low electrical conductivity of most biologically-significant polymers [12]. The incorporation of suitable functional additives containing a spatially-extended π bonding system represents a simple approach to overcome this limitation [14,15].

**Figure 1:**
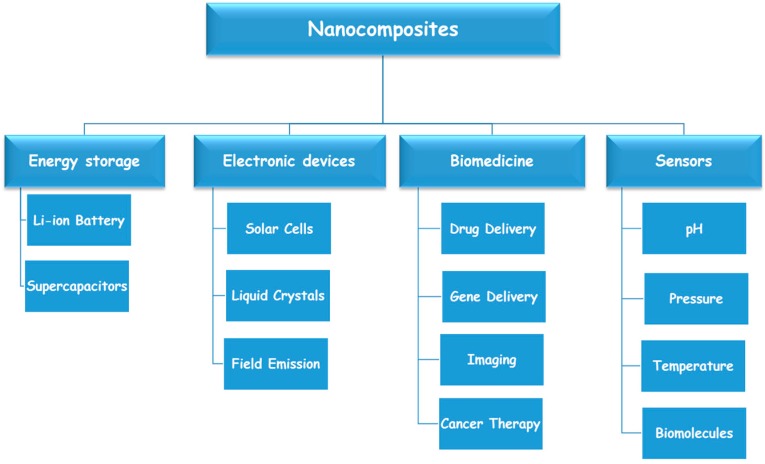
Technological applications of carbon nanohybrids.

Among the different additives, carbon nanostructures, such as carbon nanotubes (CNT) and graphene (GP), are emerging as innovative and valuable materials to fabricate electro-responsive hydrogel systems [16,17] (Figure 1).

CNT and GP are allotropes of carbon resulting from variations in covalent bonding between carbon atoms. Each allotrope has distinctive physical and chemical properties owing to the unique spatial arrangement that carbon atoms adopt [18].

The introduction of a small amount of carbon nanostructures into a polymer system allows the enhancement of some polymer properties, such as photo-conductivity [19], electro-conductivity [20], water solubility [21], mechanical and optical properties [22].

## 2. Carbon Nanotubes

Carbon nanotubes (CNT) consist of a hexagonal arrangement of sp^2^-hybridized carbon atoms (the C–C distance is about 1.4 Å) [23,24].

They can be envisioned as cylinders composed of rolled-up graphite planes with diameters in the nanometre scale [25,26]. The cylindrical nanotube usually has at least one end capped with a hemisphere of a fullerene structure [27].

Depending on the process for CNT fabrication, there are two types of CNT [28,29]: single-walled CNT (SWNT) and multiwalled CNT (MWCNT). SWCNT consist of a rolled single graphene layer, whereas MWCNT consist of two or more concentric cylindrical shells of graphene sheets coaxially arranged around a central hollow core with van der Waals forces acting between adjacent layers. The interlayer separation of the graphene layers of MWCNT is approximately 0.34 nm on average, each one forming an individual tube and the outer diameter ranging from 2.5 to 100 nm, while for SWCNT, this value ranges from 0.6 to 2.4 nm [30].

**Figure 2:**
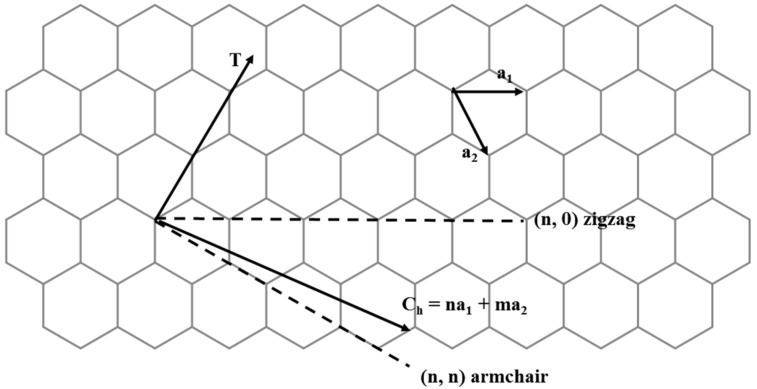
Chirality features of carbon nanotubes.

According to the rolling angle of the graphene sheet, CNT have three different chiralities: armchair, zigzag and chiral (Figure 2).

The tube chirality is defined by the chiral vector, **C**_h_ = *n***a_1_** + *m***a_2_**, where the integers (*n*, *m*) are the number of steps along the unit vectors (**a_1_** and **a_2_**) of the hexagonal lattice [31]. Using this (*n*, *m*) naming scheme, the three types of orientation of the carbon atoms around the nanotube circumference are specified. If *n* = *m*, the nanotubes are called “armchair”. If *m* = 0, the nanotubes are called “zigzag”. Otherwise, they are called ‘‘chiral”. The chirality of nanotubes has a significant impact on their transport properties, particularly on the electronic properties. For a given (*n*, *m*) nanotube, if (2*n* + *m*) is a multiple of three, then the nanotube has a metallic behaviour; otherwise, the nanotube is similar to a semiconductor. For MWCNT, since they contain a multi-layer of graphene and each layer can have different chiralities, the prediction of their physical properties is more complicated than that of SWCNT [23].

Within the realm of biotechnology, CNT have been utilized for many different applications [32], including platforms for ultrasensitive recognition of antibodies [33], as nucleic acid sequencers [34] and as bioseparators [35], biocatalysts [36] and ion channel blockers [37] for facilitating biochemical reactions and biological processes [38]. Towards nanomedicine, CNT have been utilized as scaffolds for neuronal and ligamentous tissue growth for regenerative interventions of the central nervous system [39] and orthopaedic sites [40], substrates for detecting antibodies associated with human autoimmune diseases with high specificity [41,42] and carriers of contrast agent magnetic resonance imaging [43,44]. When coated with nucleic acids (DNA or RNA), vaccines and proteins, CNT have been shown as effective substrates for gene sequencing and as gene and drug delivery vectors to challenge conventional viral and particulate delivery systems [45,46,47,48,49,50,51,52].

It should be noted that the CNT outstanding reactivity, due to their enormous surface area and achieved by their infinitesimal size, serves as their best merit, on the one hand, but also as their worst attribute, on the other, especially when they enter humans and others living creatures’ bodies [53,54,55,56], even if the exact mechanisms regulating their toxicity are still to be understood [57,58,59].

According to the WHO (World Health Organisation) definition, CNT possess a fibre-like structure, and the morphological similarity to asbestos fibres is the major concern in public health [60]. It was proven that exposing the mesothelial lining of the body cavity of mice (a surrogate for the mesothelial lining of the chest cavity) to long MWNT, an asbestos-like nanotube, length-dependent pathogenic processes are observed. These processes include inflammation and the formation of lesions known as granulomas [61]. In addition, CNT carcinogenic properties, causing mesothelioma at a high rate in intact male rats, have also been reported [62,63].

Since most of the toxicological concerns of CNT are related to their water insolubility or near insolubility [64], several studies have hence been performed aiming to prepare derivatives of highly functionalized CNT with reduced toxic effects [65]. CNT, in the pristine form, invariably exist as aggregates or bundles that are tightly bound by hydrophobic interactions between the sp^2^ carbon tube shells [66].

When dealing with the functionalization of CNT, a distinction must be made between covalent and non-covalent functionalization. Covalent functionalization is based on the covalent linkage of functional entities onto the nanotube’s carbon scaffold. It can be performed at the termini of the tubes or at their sidewalls. Direct covalent sidewall functionalization is associated with a change of hybridization from sp^2^ to sp^3^ and a simultaneous loss of conjugation. Defect functionalization takes advantage of chemical transformations of defect sites already present. Defect sites can be the open ends and holes in the sidewalls, terminated, for example, by carboxylic groups, and pentagon and heptagon irregularities in the hexagon graphene framework. Oxygenated sites, formed through oxidative purification, have also to be considered as defects [67,68].

A non-covalent functionalization is mainly based on supramolecular complexation using various adsorption forces, such as van der Waals’ and π-stacking interactions. All of these functionalizations are exohedral derivatizations. A special case is the endohedral functionalization of CNT, *i.e.*, the filling of the tubes with atoms or small molecules [69].

Among others, surface modification of CNT may be realized either by covalent or non-covalent bonding (wrapping) of polymer molecules on the surface of CNT [70].

### 2.1. Non-Covalent Wrapping Techniques

Most of the CNT-hydrogel hybrids are synthesized by non-covalent wrapping techniques involving coating with polymeric materials [71]. The wrapping techniques benefit from being non-destructive and efficient [72], but some of the best dispersants are also toxic to living cells. Different biocompatible and bioactive polymeric materials have been proposed as valuable wrapping agents [73,74], including polymeric surfactant (e.g., Pluronic F127 [75]), oligopeptides and natural extracellular matrix proteins (e.g., gelatine, collagen, elastin and laminin) [76].

For preparing non-covalent CNT-hydrogel hybrids, the employed approaches involve the use of both pristine and preliminarily-functionalized CNT.

The first approach is based on the surface properties of CNT: the sp^2^ bonded graphene structures at the sidewalls contain highly delocalized π electrons, which can form functionalized CNT with other π electron-rich compounds (e.g., conducting polymers) through π–π interactions. This organic functionalization method does not destroy the intrinsic structures of CNT and gives structurally intact CNT with functionalities, and thus, the unique properties of the CNT surface are transferred to the final nanohybrids. Recently, the potential interaction between the highly delocalized π-electrons of CNT and the π-electrons correlated with the lattice of the polymer skeleton has generated much interest and provided the motivation for studying the optical and electronic properties of composites of CNT and π-conjugated polymers [70].

On the other hand, a pre-functionalization could help with enhancing the interaction between CNT and polymer counterparts to optimize their dispersion in the hydrogel networks.

Many studies carried out on CNT/polymer composites [77] show that three main factors greatly influence the reinforcement of the CNT/polymer composite: (1) a good dispersion of the CNT in polymer matrix [78,79]; (2) a strong interfacial bonding between CNT and polymer [80]; and (3) a good alignment of the CNT in polymer matrix [81].

### 2.2. Covalent Functionalization of the CNT Surface

Alternative synthetic strategies consist of the preparation of a composite material where the CNT are covalently bound to the network.

Many techniques, including esterification, “click” chemistry, layer-by-layer self-assembly, radical polymerization, anionic coupling, supercritical CO_2_-solubilized polymerization, γ-ray irradiation, reversible addition fragmentation chain-transfer polymerization, ring-opening polymerization and atom transfer radical polymerization, have been employed to functionalize CNT with polymers [82].

**Figure 3:**
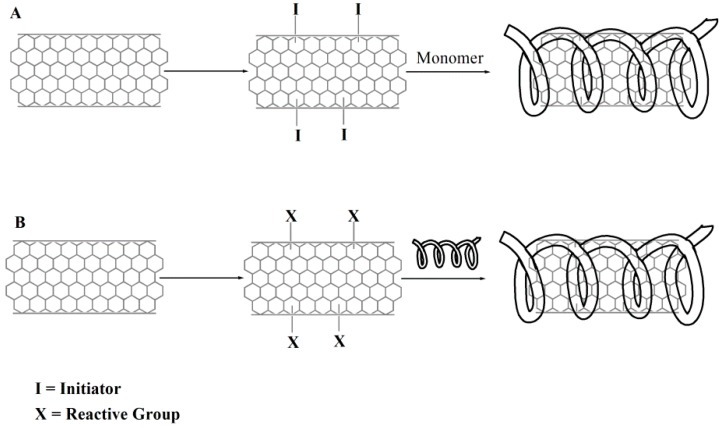
Schematic representation of the “grafting from” (**A**) and “grafting to” (**B**) approaches for CNT functionalization.

The main approaches for the fabrication of covalent CNT-polymer nanohybrids can be classified into “grafting from” and “grafting to” [83] (Figure 3).

The “grafting to” approach involves pre-formed polymer chains reacting with the surface of either pristine or pre-functionalized CNT. The structure of CNT can be used to explain the rationale of this approach. The curvature of the carbon nanostructures imparts a significant strain upon the sp^2^-hybridized carbon atoms, and as a consequence, the energy barrier required to convert these atoms to sp^3^ hybridization is lower than that of the flat graphene sheets, making them susceptible to various addition reactions. The “grafting to” method onto CNT defect sites means that the readymade polymers with reactive end groups can react with the functional groups on the nanotube surfaces. An advantage of the “grafting to” method is that preformed commercial polymers of controlled molecular weight and polydispersity can be used. The main limitation of this technique is that initial binding of polymer chains sterically hinders the diffusion of additional macromolecules to the CNT surface, leading to a low grafting density. Furthermore, only polymers containing reactive functional groups can be used [84]. The “grafting from” approach involves the polymerization of monomers from surface-derived initiators on CNT. These initiators are covalently attached using the various functionalization reactions developed for small molecules. The advantage of the “grafting from” approach is that the polymer growth is not limited by steric hindrance, allowing high molecular weight polymers to be efficiently grafted. In addition, nanotube-polymer composites with quite high grafting density can be prepared. However, this method requires a strict control of the amounts of initiator and substrate, as well as accurate control of polymerization conditions. Moreover, the continuous π-electronic properties of CNT would be destroyed by acid oxidation (required for initiator linkage). As a result, compared to the “grafting from”, the “grafting to” approach has much less alteration of the structure of CNT [85]. A further modification of the “grafting from” approach involves the direct polymerization of suitable monomers around the CNT surface, with the subsequent covalent addition to change the carbons from sp^2^ to sp^3^ hybridization [83].

### 2.3. Non-Covalent CNT-Hydrogels in Drug Delivery

As stated before, several wrapping polymers have been proposed for the preparation of non-covalent CNT composites (Table 1).

**Table 1:** Electro-responsive carbon nanohybrids discussed in this review.

Application	Composition		Synthetic Approach	Ref.
	Carbon Portion	Polymeric Portion		
**Conducting material**	SWNT	PMMA	Melt processing	[86]
		PMMA	Coagulation	[87]
	CNT	PVP	Wrapping	[96]
	GO	PDMAEMA	ATRP	[134]
**Biomedical device**	MWNT	Pluronic F127	Wrapping	[88]
		Gelatine	Wrapping	[88]
		PVP/PVA	Wrapping	[95]
		P(AAm-*co*-EBA)	Thermal polymerization	[100]
	SWNT	PPy/PEGDA	Redox/Thermal polymerization	[102]
	CNT	P(NIPAAm-*co*-MEBA)	Dispersion	[97]
	GO	Cyclodextrin-dye complex	Mixing	[140]
		PS	PEP	[154]
	rGO	SAP and IBP	Mixing	[144]
**Drug delivery system**	MWNT	PVA/PAA	Electrospinning	[98]
		IPN(PEO/PPETA)	Photo-polymerization	[101]
		P(Gelatine-*co*-NaMA-*co*-EBA)	Thermal polymerization	[104]
		P(MAA-*co*-EGDMA)	Thermal polymerization	[107]
		P(MAA-*co*-EBA)	Thermal polymerization	[109,110]
		P(Gelatine-*co*-AAm-*co*-EBA)	Photo-polymerization	[111]
		Cellulose	Mixing	[113]
	CNT	PVA	Mixing	[112]
	GO/rGO	α-Cyclodextrin/Pluronic F-127	Mixing	[141]
	GO	KGM/SA	Mixing	[142]
		RGD-chitosan	Mixing	[143]
		Chitosan	Condensation	[148]
		PPy	Electro-polymerization	[149]
		PEI	Condensation	[150]
		PVCL	ATRP	[152]
		IPN(PEO/NIPAAm-*co*-MEBA)	Condensation	[153]
	rGO	PVA	Mixing	[146]
		JPT/PEGDE	Radical polymerization	[155]
**Electrical sensor**	MWNT	Gelatine	Wrapping	[89,90]
		C8-LPEI/EGDGE	Mixing	[99]
	SWNT	PEGDA	Photo-polymerization	[103]
**Photosensitisers release**	GO	HA	Condensation	[151]

CNT = carbon nanotubes; MWNT = multi-walled carbon nanotubes; SWNT = single-walled carbon nanotubes; GO = graphene oxide; GP = graphene; rGO = reduced graphene oxide; AAm = acrylamide; C8-LPEI = octyl modified linear polyethylenimine; EBA = *N*,*N*'-ethylenebisacrylamide; EGDMA = ethylene glycol dimethacrylate; EGDGE = ethylene glycol diglycidyl ether; HA= hyaluronic acid; IBP = hyperbranched polymer; IPN = interpenetrating polymer network; JPT= Jeffamine polyetheramine; KGM= konjac glucomannan; MAA = methacrylic acid; NaMA = sodium methacrylate; NIPAAm = *N*-isopropyl-acrylamide; MEBA = *N*,*N*'-methylenebisacrylamide; PDMAEMA = poly-2-(dimethylamino)ethyl-methacrylate; PEGDA = polyethylene glycol diacrylate; PEGDE = polyethylene glycol diglycidyl ether; PEI = poly-ethylenimine; PEO = polyethylene oxide; PMMA = polymethylmethacrylate; PPETA = polypentaerythritol triacrylate; PPy = polypyrrole; PS = polystyrene; PVA = polyvinyl alcohol; PVCL = poly-*N*-vinyl caprolactam; PVP = polyvinylpyrrolidone; SA= sodium alginate; SAP= superadsorbent polymer; ATRP = atom transfer radical polymerization; PEP = Pickering emulsion polymerization.

Solvent casting followed by melt mixing and coagulation methods were proposed to produce SWNT/poly(methyl methacrylate) nanocomposite fibres with enhanced elastic modulus, thermal stability and conductivity [86,87].

Pluronic F127 and gelatine were employed as wrapping materials in MWNT nanohybrids useful in the biomedical field [88]. MWNT/gelatine composites were prepared by dispersion of MWNT through ultrasonication in an aqueous medium with the anionic surfactant sodium dodecyl sulphate [89]. The key result of the study is the possibility to couple the advantages of both the nanostructure and protein. CNT act as reinforcing agents, considerably improving the structural and mechanical properties of the gelatine [90], while gelatine facilitates the dispersion of the carbon structures allowing, at the same time, the use of the final nanohybrid in biomedicine, where the protein is widely employed as a plasma expander, wound dressing, adhesive agent and controlled drug delivery system [91,92,93,94]. The water affinity of the nanohybrids was also found to be closely related to the CNT content: an increase in the CNT content resulted in a decrease in the swelling properties, while the stability and reversible behaviour upon exposure to direct current are considerably enhanced when compared to the protein alone, which underwent remarkable erosion processes.

Synthetic polymers can also be useful for the obtainment of composites for pharmaceutical and biomedical purposes. The coating of MWNT with poly(vinyl alcohol) allows the obtainment of versatile composites for different applications, including biosensors, artificial cartilage substitutes and drug delivery systems [95]. The formation of CNT/poly(vinyl alcohol) composite hydrogels without using chemical treatments of CNTs and chemical crosslinking agents in gel formation would enhance the mechanical and electrical properties of the hydrogels, meanwhile maintaining the good biocompatibility of both nanotubes and poly(vinyl alcohol) hydrogels.

Poly(vinyl pyrrolidone) is another valuable polymer employed for the synthesis of hybrid hydrogels where CNT are uniformly dispersed. Furthermore, in this case, the strong interface between the nanotubes and matrix allows the preservation of the CNT pristine surface structure and the obtainment of exceptional mechanical and physical properties [96]. The surfactant poly(vinyl pyrrolidone) in cooperation with MWCNT demonstrated an important synergistic effect on the formation and performance of the composite hydrogels.

A different approach is based on the introduction of CNT into a thermally-sensitive poly(*N*-isopropylacrylamide) hydrogel matrix to fabricate hybrid hydrogels exhibiting an electric current sensitivity resulting from electro-thermal conversion. When an external electric field is applied, the electro-thermal conversion takes place to raise the local temperature of the composite beyond the lower critical solution temperature (around 32 °C) of poly(*N*-isopropylacrylamide) hydrogel. Here, an indirect and contact-type electric sensitivity is reached, while when polyelectrolyte hydrogels are employed, they always require being placed between two electrodes in a non-contact status [97].

As stated before, other strategies for the preparation of hybrid composites required a pre-functionalization of the CNT surface.

A preliminary surface modification of MWNT can be carried out by oxyfluorination to insert functional groups on their hydrophobic surface for improving the dispersion and the compatibility with a polymer matrix composed of poly(vinyl alcohol)/poly(acrylic acid) hydrogel. Thus, an electro-responsive transdermal drug delivery system was prepared by electrospinning [98]. A uniform distribution of the oxyfluorinated MWNT in the nanofibers was found to be crucial to the electro-responsive swelling and drug releasing behaviours. The amount of released drug increased by increasing the content of MWCNT, by using an oxyfluorination with higher oxygen content and by applying the highest electric voltage. The nanocomposite showed more than 80% cell viability, enough to be used as a non-toxic and biocompatible material.

In another study, Meredith *et al.* suggested the application of a CNT composite electrode hydrogel as a glucose biosensor and biofuel [99]. The mechanism at the basis of idea is the facile oxidation of nicotinamide cofactors, such as NADH, by azine dyes inserted inside the electrode. Azine dyes, carbon nanotubes, polymer hydrogels and enzymes were incorporated in composite electrodes in one single mixing step. The electro-polymerized composite electrodes were found to display the ability to electrocatalytically oxidize NADH more efficiently than a control electrode prepared without CNT.

### 2.4. Covalent CNT-Hydrogel in Drug Delivery

By inserting various amounts of MWNT (0.001, 0.002 and 0.01 wt %) in a radical polymerization of acrylamide and bisacrylamide, composite hydrogels with tuneable physico-chemical properties were synthetized and proposed for different applications, including artificial muscles, drug delivery, robotics and micropumps. The different amounts of CNT affect many properties, such as electro-responsive behaviour, water uptake and surface roughness, while not significantly influencing the electrical conductivity due to the lack of alignment of nanotubes in the polymeric network. In addition, water uptake depends on the drying method employed to recovery hydrogels [100].

A semi-interpenetrating polymer network, reinforced with MWNT and obtained by photo-polymerization of pentaerythritol triacrylate and polyethylene oxide, was proposed for the preparation of an electro-sensitive transdermal drug delivery system for ketoprofen [101]. The MWNT amount in the electrospun fibres confers remarkable electrical conductivity up to a MWCNT/PEO ratio of 10 wt %, while no further significant increase is recorded above this value.

The materials show a high cell viability (over 85%) on mouse fibroblasts, confirming their high biocompatibility. The *in vitro* drug diffusion experiments, tested by vertical Franz-type diffusion cells, were performed at various electric voltages (0, 5, 10 and 15 V) using both hybrid hydrogels and blank materials not containing CNT. For both materials, the amount of released drug significantly increases within the applied voltage. Furthermore, the release is greater for the hybrid than for the blank fibres, because the electrical stimulation results in a partial dissolution of polyethylene oxide in the semi-interpenetrating polymeric network, creating empty spaces, which represent the route for the drug diffusion.

An alternative methodology to improve the electrical conductivity and mechanical strength of the hydrogels without significant reduction in the swelling properties of SWNT composites was obtained through the interfacial polymerization of SWNT, polypyrrole and poly(ethylene glycol) diacrylate [102]. The ternary SWNT/polypyrrole (PPy)/ polyethylene glycol diacrylate (PEGDA) hydrogel possesses improved electrochemical performance, enhanced mechanical toughness, compactness and homogeneity of the surface structure than the binary hybrid hydrogel of SWNT/PEGDA previously reported by the same authors [103]. This conductive composite hydrogel could be applied in the field of implantable devices, including neural prosthetic devices, biosensors and bioelectronic arrays.

Spherical hybrid hydrogels composed of gelatine and MWNT were synthesized by emulsion polymerization in the presence of sodium methacrylate and *N*,*N*'-ethylenebisacrylamide and proposed as drug delivery microspheres for the electro-responsive release of diclofenac sodium salt [104]. Emulsion polymerization allows producing spherical particles with a narrow size distribution and dispersing designated functional molecules (e.g., MWNT) into the polymerization feed. With this technique, the gelatine can act as a macromolecular co-crosslinker in the synthesis of spherical gels (average diameters around 100 µm) suitable for delivering a therapeutic in response to pH and/or temperature modifications without any preliminary modification of the protein structure [105,106].

This straightforward procedure allows obtaining composite materials in a one-step process showing considerable advantages from both a synthetic and an application point of view; it is a single step and easily scalable process, and the intrinsic properties of either CNT or protein are not affected, but totally retained in the final composite.

The swelling properties confirm the polyelectrolyte behaviour of hydrogels, and the application of an external electric field (at pH 7.4) caused a significant reduction of their swelling degree as a consequence of a built-in osmotic pressure. Drug release experiments demonstrated the ability of the composites to control the release of drug over time. The electric stimulation resulted in a further increase of the release (+20%) in MWNT-containing materials.

The synthesis of molecularly-imprinted electro-responsive nanohybrids was reached by precipitation polymerization of methacrylic acid and ethylene glycol dimethacrylate in the presence of CNT [107]. Diclofenac sodium salt was employed as a template molecule, and its release profile was investigated to elucidate the ability of nanohybrids to release the template in response to 20-V DC voltage.

An interesting upgrade of the application of hybrid hydrogels as electro-responsive delivery systems is the possibility to reach pulsatile release profiles by the on/off switching of an applied electric field [108]. Electro-responsive MWNT-poly(methacrylic acid) hydrogel hybrids synthetized by *in situ* radical polymerization were proposed for “on demand” drug delivery [109]. Experiments, carried out employing radio-labelled sucrose as the model hydrophilic drug molecule showed a pulsatile release profile upon the on/off application of the electric field, and a drug release from the hybrid gels up to 70% was achieved after 80 min. The release of a model drug upon the application of a DC electric field was demonstrated *in vivo* following sub-cutaneous implantation of the hybrid hydrogels in a mouse model. This drug delivery device produces greater amounts of released drug from gel with a high MWNT content; however, the amount of drug released between each cycle of on/off electrical stimulation was not reproducible and decreased dramatically after the second electrical stimulation. Producing an adherent hybrid hydrogel is not trivial, as the reversible swelling and de-swelling of the polymer matrix upon electrical exposure can lead to appreciable levels of shear stress, which can result in structural damage. The same authors also studied the mechanisms by which MWNT improve the electrical, mechanical and thermal properties of the composite [110]. The presence of MWNT within the hydrogel matrix affected the mechanical properties of the hydrogel by decreasing the pore size and therefore the swelling/de-swelling of the gels. The damage to the hybrid gel surfaces after electrical stimulation and the loss of the pulsatile release profile at high cross-linker concentrations suggested that the mechanism of drug release involved a compression effect and magnified the stress on the polymeric network as a result of the electrical properties of MWNT.

Electro-responsive hybrid hydrogels, able to precisely modulate the release of therapeutics by the application of an external voltage, were proposed by the covalent insertion of MWNT into acrylamide-*co*-ethylene/bis-acrylamide hydrogels [111]. In this study, MWNT act as functional elements, responsible for both drug interaction and the electro-responsivity. The release/retention profile was closely related to the charge, with anionic drugs (diclofenac sodium salt) released at 12 V from the negatively-charged MWNT outer surface, and cationic drugs (ciprofloxacin) were retained at 12-V conditions and quickly released in off conditions (0 V). Hydrogel properties were found to depend strongly on the MWNT content, with the possibility to reach an on demand delivery by switching the external voltage on/off.

Different kinds of CNT hybrid materials were proposed as an electric interface between neural tissue and electrodes in the development of implantable devices for continuous monitoring and functional stimulation of the central nervous system in terms of electro-activity, biocompatibility and long-term stability for chronical application. To engineer an interface possessing these merits, a polymeric hydrogel based on poly(ethylene glycol) diacrylate and SWNT was employed to fabricate a hybrid hydrogel via a covalent anchoring strategy, *i.e.*, self-assembly of cysteamine followed by Michael addition between cysteamine and poly(ethylene glycol) diacrylate [103]. According to the authors, this hybrid hydrogel provides a favourable biomimetic microenvironment for cell attachment and growth due to its inherent biocompatibility and good adhesion to the electrode substrate.

More recently, Choi *et al.* synthesized CNT-incorporated polyvinyl alcohol (PVA)-based hydrogels by an electro-click reaction, which was controlled by an electrochemically-generated Cu(I) catalyst [112]. Results showed that CNT-incorporated hydrogel films were deposited faster than hydrogel films in the absence of CNT, as the embedded carbon nanotubes provided a larger electrochemically-active area. The release behaviour of tetracycline was enhanced under negatively-biased potential at pH 8 due to charge–charge repulsion.

Novel hybrid hydrogels composed of sodium alginate, bacterial cellulose and MWNT were synthesized using CaCl_2_ as a crosslinking agent and proposed as a pH and electric field dual-stimulus responsive drug delivery system [113]. The releasing profile of the drug from the hybrid hydrogels was found to be dependent on the applied electric current strength at neutral pH and selective to the pH value of the surrounding acidic or alkaline environment, with little difference in the presence of the applied electric voltage. Drug release was mainly affected by the pH-induced protonation and deprotonation of sodium alginate and was enhanced under electric voltage application.

## 3. Graphene

The atomic structure of graphite is characterized by the multiple stacking of one atom-thick sheets formed by carbon atoms arranged in a hexagonal lattice. The isolated two-dimensional crystal structures made of single atomic layers of graphite are called “graphene”. The existence of single graphene sheets had been discussed in theory more than 50 years ago [114]. Yet, the existence of two-dimensional, atomically-thin crystal materials was considered physically impossible [115]. In 2004, a single sheet of graphene was isolated and characterized by Novoselov and Geim [116]. Since then, research on graphene has been increasing almost exponentially, attracting the interest of various scientific fields [117].

Interest in monoatomic graphene sheets also drew attention to other carbon-based materials that are now examined under a different light. The most notable of them is graphene oxide, *i.e.*, graphene sheets derivatized with oxygen-containing functional groups. By analogy to graphite and graphene, graphene oxide is the “building block” of graphite oxide. More particularly, it is the result of graphite’s oxidation under acidic conditions, first described by Brodie *et al.* more than 150 years ago [118]. Today, the most popular method for the production of graphene oxide is based on the principle first introduced by Hummers and Offeman (commonly referred to as the Hummers method) that involves the oxidation of graphite by potassium permanganate in concentrated sulphuric acid [119].

Graphene is composed almost entirely of sp^2^-hybridized carbon atoms and their electrons participate in aromatic conjugated domains. In graphite, van der Waals forces keep graphene sheets tightly together. Even in the case of single graphene sheets synthesized *de novo* (for example, through chemical vapour deposition of carbohydrates on a metal catalyst), the apolar nature of the carbonaceous material makes them highly hydrophobic [120]. On the other hand, the oxidation of graphite to graphite oxide loosens its firmly-packed graphene sheets. This occurs due to the random introduction of carbonyls, hydroxyls and epoxides on the planar surfaces and edges of the carbon sheets. Graphene oxide sheets can then be exfoliated from graphite oxide particles through ultra-sonication [121].

Graphene oxide has important potential applicability in drug delivery due to the presence of functional groups (epoxy, hydroxyl, carboxylic groups), conjugation systems, large surface area, low cytotoxic effects and low costs [122]. The presence of these functionalities decorating the basal planes and edges of GP layers significantly revises the van der Waals interactions between graphene sheets, thus conferring desirable dispersibility [123]. Furthermore, these functional groups, together with the high surface area and π-conjugated structure of GP, allow conjugation with a number of substances through both covalent and non-covalent modification techniques [124].

### 3.1. Graphene-Hydrogel Hybrids

Graphene can be functionalized via non-covalent binding, such as π–π stacking, cation-π and van der Waals interactions [125,126,127]. In contrast to covalent modifications, non-covalent ones are considered to have less impact on the conjugated structure and the mechanical and electrical properties of graphene.

Recently, a considerable number of works aimed to enhance the properties of graphene by chemical modification, obtaining new graphene derivatives [128,129,130]. The grafting of polymer chains onto graphene is proposed as an efficient way to improve the properties of graphene, such as its solubility [131], interfacial interactivity with target matrix [132] and electronic properties [133].

Atom transfer radical polymerization is one of the first used approaches to graft initiator molecules onto GO sheets and, subsequently, to synthesize *in situ* poly[2-(dimethylamino)ethyl methacrylate] chains on their surface [134]. As reported for CNT, this approach is unable to control the molecular weight and architecture of a resultant polymer, and the reaction conditions are also severely restricted by graphene solubility in a solvent [135]. Thus, also in the GP case, the “grafting to” approach seems to be a promising and simple way to functionalize GP with well-defined polymer chains, and several polymer-grafted GP nanocomposites have been synthesized using different coupling techniques [136,137,138,139].

### 3.2. Non-Covalent Graphene-Hydrogel Hybrids in Drug Delivery

A valuable approach for the preparation of hybrid hydrogels composed of GO and GO derivatives is the non-covalent inclusion into supramolecular structures, like cyclodextrins. This confers high elastic behaviour at elevated temperature to the resulting supramolecular hydrogels [140]. A key example of this concept was developed by a preliminary stabilization of GO in solution by the surfactant Pluronic F-127 and a subsequent inclusion into α-cyclodextrins. By this approach, hydrogels for potential systemic administration were prepared, with the key advantages of improving the ability of hydrogels to bind doxorubicin molecules and better controlling the release in comparison to a native hydrogel prepared in the absence of GO [141].

A similar approach involves the insertion of GO into composite hydrogels based on natural polysaccharides, such as konjac glucomannan and sodium alginate [142]. As expected, the GO nanosheets were found to significantly influence the micromorphology, swelling ability and drug loading of the composite hydrogel. Interestingly, *in vitro* release studies showed that the burst release phenomenon could be avoided and that an excellent pH-sensitive system could be achieved.

Efficient GO-hydrogel hybrids have been synthesized by the non-covalent coating of GO with cyclic RGD-modified chitosan. The nanohybrid was proposed as a drug delivery system for the targeted delivery of doxorubicin to hepatocellular carcinoma, with the GO counterpart conferring efficient loading properties, while the GO-chitosan hydrogen bonding interactions allow the selective drug release in the acidic tumour environment due to the pH-responsive behaviour. Furthermore, the nanocomposite is able to recognize hepatoma cells and to promote drug uptake by the cells, especially for those overexpressing integrins, as confirmed by cellular uptake and proliferation studies [143].

A similar non-covalent approach was employed for the preparation of a flexible, electrically-conducting hydrogel based on commercial superabsorbent polymer and hyperbranched polymer. Here, the incorporation of reduced graphene oxide (rGO) resulted in excellent electrical self-healing properties and water-absorption reusability [144].

Yi Wang and co-workers reported on the fabrication of flexible self-supporting hybrid films composed of GO intercalated with Mg–Al layered double hydroxide via a solvent evaporation process and proposed it as a tool for the delivery of benzylpenicillin [145]. The key finding of this study is the possibility to tailor the drug release by modification of the film composition. More interestingly, the results of antibacterial tests highlight that hybrid films can inhibit bacterial growth with key advantages from an application point of view.

Hybrid hydrogel membranes composed of reduced graphene oxide nanosheets and a poly(vinyl alcohol) matrix were proposed as an electrically-responsive drug release system for the anaesthetic drug lidocaine hydrochloride by Heng-Wen Liu *et al.* [146]. The presence of rGO in the nanohybrids was found to act as a physical barrier to inhibit the drug release, while the exposure to an electrical stimulus highly enhances the release.

### 3.3. Covalent Graphene-Hydrogel Hybrids in Drug Delivery

He *et al.* immobilized different kinds of functional moieties or polymers onto GP sheets via nitrene chemistry to obtain highly-engineered composite materials [147]. As a result, the functionalized graphene nanosheets, electrically conductive and dispersible in different solvents, remained in individually separated single layers, while the introduced functional groups can be further modified by differently chemical reactions for the fabrication of different kinds of graphene oxide-based hybrids.

Kumar Rana *et al.* prepared a covalent nanohybrid composed of chitosan and GO by condensation reaction [148]. Specifically, the carboxylic acid groups of GO were converted into acyl chlorides, and then, the conjugation to chitosan was achieved. The composite was proposed as a delivery device for ibuprofen and 5-fluorouracil with high efficiency due to its ability to penetrate into the cells. The releasing profile was found to be closely dependent on the ionization properties of the two drugs, with the release reaching a faster rate when the drugs are in the ionized form. Electrospinning methods were employed by Weaver *et al.* [149] for the preparation of GO nanohybrids able to act as drug delivery systems for the controlled release of the anti-inflammatory drug dexamethasone in response to electrical stimulation. The key finding of the study was the possibility to finely modulate the properties, such as the stability and dosing, of the GO nanocomposite release platform, which is proposed as a valuable tool for advanced drug delivery technologies. More in detail, polypyrrole was employed as the conducting element, while the GO nanosheets acted as nanocarriers, improving the amount of drug loaded into and released from the nanocomposite film. The authors stated that the system can be tuned according to various needs, making it a valuable tool for both therapeutic and research applications. The nanocomposite film, indeed, exhibited a linear release profile persisting over several hundred stimulations, and this is a clear indication that it could be used for long-term drug-release applications requiring repeated dosing over time.

The binding properties of the GO surface were explored for the preparation of a gene delivery vector [150]. In this approach, low-molecular weight branched polyethylenimine was employed as a cationic gene carrier, with GO acting as a fluorescence reagent probe. According to the authors’ hypothesis, the excellent photoluminescence activities of the GO-based nanoconstruct will definitely merit further attention in the development of more sophisticated carrier systems that could serve both as a gene delivery vector and bioimaging tool.

GO can also act as a high loading effector for photosensitiser agents, as proven by Li *et al.* [151]. Here, a hyaluronic acid-GO conjugate was proposed as a cancer cell target and photoactivity switchable nanoplatform for photodynamic therapy. The conjugate was prepared through a multi-step synthetic strategy involving at first the chemical conjugation of modified hyaluronic acid and fractionated GO sheets (below 100 nm to enhance releasing efficiency). Subsequently, a photosensitiser was loaded into the GO sheet via π–π stacking and hydrophobic interactions. The photoactivity of the photosensitiser adsorbed on the nanocarriers was mostly quenched in aqueous solution to ensure biocompatibility, but was quickly recovered after the release from nanocarriers upon cellular uptake. The photodynamic therapy was remarkably improved, resulting in efficiencies 10-times higher than that of the free photosensitiser.

The loading properties of the GO surface via π–π stacking and hydrophobic interactions were also exploited for the preparation of pH-sensitive drug delivery devices with poly-*N*-vinyl caprolactam acting as the polymer counterpart conferring high solubility and stability in water and physiological solutions to GO [152]. GO was inserted via atom transfer radical polymerization, and the resulting composite was proposed for the intracellular delivery of the anticancer drug camptothecin with high releasing efficiency. The composite showed a high loading ratio, and the loaded drug is quickly released at a reduced pH (typical of the tumour micro-environment). In addition, the presence of poly-*N*-vinyl caprolactam allows a targeted and temperature-dependent delivery rate. The authors demonstrated that the pure nanocargo alone was almost non-toxic, whereas the nanohybrid showed strong potency against cancer cells, due to a cellular-uptake mechanism of energy-dependent endocytosis.

Thermo-sensitive releasing behaviour by GO nanohybrids was also reached by the covalent immobilization of thermo-responsive polymer nanoparticles on functionalized GO nanosheets. In the synthetic procedure, thermo-sensitive nanoparticles were first synthesized by free radical polymerization, and GO nanosheets were non-covalently modified with a bi-functional linker able to provide reactive sites for the subsequent binding to the polymer. Then, Adriamycin was loaded with high efficiency and the whole system tested in suitable mouse models with interesting results [153].

In a different approach, the Pickering emulsion polymerization method was proposed for the fabrication of core-shell-structured polystyrene microspheres containing GO units [154]. Here, GO shows the double activity of coating the polymer microspheres and imparting electro-responsive properties to the polymer composite suspension under an applied electric field.

An electro-conductive hydrogel system was prepared by incorporation of rGO into Jeffamine polyetheramine and polyethylene glycol diglycidyl ether by a one-step polymerization method [155]. The obtained hybrid hydrogels showed enhanced mechanical and electrical properties depending on rGO content and were investigated as a delivery device for methyl orange. A significant reduction in the passive release of the dye was observed by incorporating rGO, while upon electrical stimulation, the release rate and dosage could be tuned through variation of the percentage *w*/*w* of rGO, as well as of the polarity and amplitude of the applied electric potential.
